# Serum miR-486-5p as a diagnostic marker in cervical cancer: with investigation of potential mechanisms

**DOI:** 10.1186/s12885-017-3753-z

**Published:** 2018-01-09

**Authors:** Chunmei Li, Xiaojiao Zheng, Wei Li, Fumao Bai, Jianxin Lyu, Qing H. Meng

**Affiliations:** 1 Key Laboratory of Laboratory Medicine, Ministry of Education of China, Zhejiang Provincial Key Laboratory of Medical Genetics, School of Laboratory Medicine and Life Sciences, Wenzhou Medical University, Wenzhou, Zhejiang 325035 China; 2 Department of Obstetrics-Gynecology, The First Hospital of Ningbo, Ningbo, Zhejiang 315035 China; 3 Department of Laboratory Medicine, The University of Texas MD Anderson Cancer Center, Houston, TX 77030 USA

**Keywords:** Cervical cancer, miR-486-5p, Pten, PI3K/Akt

## Abstract

**Background:**

Around the world, cervical cancer is the fourth most common cancer among women. MicroRNAs (miRNAs) and agents that target mRNAs have been introduced as novel diagnostic markers and therapeutic approaches, respectively, in cancer. MiRNA-486-5p is a candidate regulator of phosphatase and tensin homolog (PTEN) in silico, and the downregulation of PTEN in cervical cancer is not consistent with its mutation, which suggests that PTEN may be subjected to post-transcription modification moderated by miRNAs. Here, we aimed to explore whether miR-486-5p is a regulator in the development of cervical cancer through the PI3K/Akt pathway by targeting PTEN.

**Methods:**

The expression level of miR-486-5p in human cervical cancer serum and tissues were analyzed through quantitative RT-PCR. Human cervical cancer cell lines HeLa and SiHa were selected to explore the effects of miR-486-5p downregulated or overexpression on cell proliferation, migration, and invasion, respectively. Moreover, we observed the effect of miR-486-5p downregulated on tumorigenesis using HeLa cell in vivo. Besides, the relationship between miR-486-5p and PTEN were determined by dual luciferase reporter gene assay.

**Results:**

Compared to control subjects, miR-486-5p was significantly overexpressed in cervical cancer patients’ serum and tissues. Suppression of miR-486-5p expression significantly inhibited HeLa cell proliferation, colony formation, migration, and invasion, as well as tumor growth in nude mice, while miR-486-5p overexpression stimulated SiHa cell proliferation, colony formation, migration, and invasion. We also confirmed that miR-486-5p directly targeted the 3′-untranslated region of the tumor-suppressor gene *PTEN*, inhibiting its expression, and that overexpression of miR-486-5p activated the PI3K/Akt pathway.

**Conclusions:**

We conclude that miR-486-5p stimulates cell proliferation, migration, and invasion through inhibition of PTEN expression and activation of the oncogenic PI3K/Akt pathway in cervical cancer. Our findings implicate serum miR-486-5p as a novel molecular biomarker that may provide effective approaches to both diagnosis and treatment in cervical cancer.

## Background

Around the world, cervical cancer is the fourth most common cancer among women, accounting for an estimated 529,572 diagnosed new cases and 274,967 deaths each year [1]. This cancer is especially prevalent in developing countries, claiming the second highest incidence and the third highest death rate of all malignancies, [2, 3]. Conventional treatment of cervical cancer involves surgery and chemo-radiotherapy, but these therapies are generally effective only in the early stages of the disease [4], and approximately half of cases persist or recur despite these treatments [5]. No well-characterized biomarkers are available for this cancer; such biomarkers would support early diagnosis, prediction of disease progression, and outcome improvement.

MicroRNAs (miRNAs) are a sort of short (18-25 nt) noncoding RNAs that have been proved to be important post-transcriptional regulators of gene expression in cells. They bind to the 3′-untranslated region (3′-UTR) of the mRNA of a specific gene and significantly modulate gene expression, influencing many biological processes [6–8]. Aberrant miRNA expression is involved in the pathogenesis and progression of cancers [9, 10] and thus has the potential to serve as a biomarker for the disease.

MiR-486-5p has been shown to be aberrantly expressed in many cancers, including myeloid leukemia [11, 12], gastric adenocarcinoma [13], and lung cancer [14], but its function in cancer is still not fully understood. MiR-486-5p is a candidate regulator of phosphatase and tensin homolog (PTEN) in silico. The downregulation of PTEN in cervical cancer is not consistent with its mutation, which suggests that PTEN is subject to post-transcription modification moderated by a miRNA in this disease. We hypothesized that miR-486-5p is a crucial regulator in the development of cervical cancer through the PI3K/Akt pathway by targeting PTEN. In this study, therefore, we investigated the potential role and biological function of miR-486-5p in cervical cancer.

## Methods

### MiR-486-5p level in cervical cancer patients and healthy subjects

Serum samples and tissues (cancer tissues and adjacent normal tissues) were obtained from 21 patients at the First Hospital of Ningbo who had a confirmed diagnosis of cervical cancer by American Joint Committee on Cancer criteria. Control serum samples were obtained from 21 healthy donors who came for an annual check-up at the same hospital. This study was approved by the ethics committee of the First Hospital of Ningbo. Clinical details for the cancer samples are presented in Table 1.

**Table 1:** Clinicopathologic characteristics of cervical cancer patients (*n* = 21)

Characteristic	Patients, n	Cq value Mean (SD)	95% CI	Univariate analysis, *P*	Multivariate analysis, *P*
Patient age, years					
≥ 50	8	25.74 (1.34)	24.62-26.87	NS	NS
≤ 49	13	25.52 (1.33)	24.72-26.32		
Human papillomavirus					
Negative	9	25.85 (1.43)	24.75-26.94	NS	NS
Positive	12	25.42 (1.24)	24.63-26.21		
Therapies					
Chemotherapy	3	25.95 (1.21)	22.94-28.95	NS	NS
Surgery	18	25.55 (1.35)	24.88-26.22		
Histologic type					
Squamous carcinoma	14	25.85 (1.34)	25.07-26.62	NS	NS
Adenocarcinoma	7	25.12 (1.17)	24.04-26.20		

*NS* not significant

MiRNAs were extracted from serum and tissues by using the miReasy Mini kit (QIAGEN, Duesseldorf, Germany). Subsequent detection of miRNAs were carried out with Taqman probes (miR-486-5p, U6) that acquired from the Taqman miRNA assay (Life Technologies, Carlsbad, CA, USA) and TaqMan PCR Master Mix (TAKARA, Dalian, China) on the StepOne Plus Real Time PCR Detection System (Life Technologies). The expression level of miR-486-5p was assessed by the relative quantification method with *U6* as the reference gene (RQ = 2^-ΔΔCt^). The high expression of miR-486-5p in cervical cancer was verified by comparing with the controls. The correlation of miR-486-5p expression in cervical cancer patients’ serum and tissues, and the receiver operating characteristic (ROC) analysis of serum miR-486-5p expression in cervical cancer patients vs healthy subjects were analyzed by Graphpad 6.0 software.

### Cell lines and cell culture

HeLa and SiHa cervical cancer cell lines were obtained from the cell bank of the Chinese Academy of Sciences (Shanghai, China). The cell lines were authenticated by the institution by using the DNA-fingerprinting method with four paired primers: DXS52, Apo B, MD17S5, and D2S44. Cells were cultured in Dulbecco minimal essential medium (DMEM) supplemented with 10% (*v*/v) heat-inactivated fetal bovine serum (FBS; Natocor, Cordoba, Argentina) and antibiotics (100 U/mL penicillin and 100 μg/mL streptomycin) in a humidified atmosphere of 5% CO_2_ at 37 °C.

### MiR-486-5p transfection and expression in cervical cancer cells

Cervical cancer cells (2 × 10^5^ cells) were transfected with 1 μg of a plasmid expressing miR-486-5p inhibitor sponge, miR-486-5p mimic, or control miRNA (Genepharma, Shanghai, China). Transfection into cells was carried out according to the manufacturer’s instructions using Lipofectamine3000 for 48 h (Invitrogen–Life Technologies). Stable expression of each miRNA was established in the cells after 15 days of incubation in complete DMEM with blasticidin (12 μg/mL). We verified miRNA expression in the clones using real-time PCR and integrate all the successful clones for the subsequent experiments.

### Cell miRNA expression analysis

Taqman probes and primers (miR-486-5p, U6) were purchased from the Taqman miRNA assay (Life Technologies). Subsequent detection of miRNAs were carried out with the TaqMan PCR Master Mix on the StepOne Plus Real Time PCR Detection System (Life Technologies). The expression level of miR-486-5p was assessed by using the relative quantification method with *U6* as the reference gene and was verified by comparison with control cells.

### HeLa and SiHa cell proliferation, colony formation, migration, and invasion

Cell proliferation was quantified by measurement of absorbance of water-soluble tetrazolium salt at 450 nm, using the Cell Counter Kit-8 (Dojindo, Kumamoto, Japan). The ratios of the specific optical density (OD) value at different time points to the control were determined. The details were refer to our previous study [15].

Cell migration was assessed by a wound healing assay described previously [15]. In brief, transfected cells were plated in a 12-well plate until cells were 80% confluent, and three wound lines were scratched vertically in each well, perpendicular to the bottom of the well, with a 200-μL pipette tip. Then the cells were washed three times with phosphate-buffered saline solution (PBS) and incubated in growth medium containing 2% serum. Take the scratched picture using a microscope (Nikon, Tokyo, Japan) at different time point 0 h, 24 h, 48 h, 72 h, and measure the closure rate by ImagePro Plus 6.0 software.

Cell invasion was measured by chamber experiment described previously [15]. In brief, transfected cells (8 × 10^4^ cells) were seeded into a 24-well transwell plate coated with matrigel (1:10 diluted with DMEM; BD Biosciences, Franklin Lakes, NJ, USA). DMEM with 10% FBS as a chemoattractant was placed in the lower chambers. 24 h later, the chamber were fixed with 4% paraformaldehyde for 20 min and stained with crystal violet for 20 min at ambient temperature, then photographed under a microscope and calculated cells number (Nikon, Tokyo, Japan).

### Xenograft tumor model in mice

Fifteen female BALB/c nu/nu nude mice (aged 4 weeks, 18–22 g) were purchased from the Shanghai Medical Experimental Animal Care Commission (Shanghai, China). The mice were housed and manipulated in specific pathogen-free conditions and controlled light/dark cycle, temperature, and humidity. This study was approved by the Ethical Committees on Animal Research of Wenzhou Medical University. Experiments performed after 1 week feeding adaptation, and the mice were randomly divided into two groups (Mock and Inhibitor).

For each mouse, HeLa cells transfected with miR-486-5p inhibitor or control miRNA (10^7^ cells per mouse in 200 μL of serum-free medium) were injected subcutaneously into the left axilla of each mouse. Tumor growth was measured every 7 days for 21 days. On day 21, the mice were anesthetized with chloral hydrate and killed by decapitation, and their tumors were removed, measured, and weighed. The tumor tissues were fixed in 10% formalin for sectioning and histologic examination.

### Western blotting

This experiment was accordance with our previous study [15]. In briefly, cell lysates were prepared in Protein Lysis Buffer (Beyotime, Shanghai, China) according to the manufacturer’s directions. The primary antibody (1:1000 dilution) and secondary antibody (1:3000 dilution) were purchased from cell signaling technology (Cell Signaling Technology, Danvers, MA, USA). GAPDH was used as an endogenous control to normalize the protein expression data.

### Immunohistochemistry

Each step of the immunohistochemical (IHC) analysis was performed according to the manufacturer’s instructions. For antigen retrieval, slides mounted with formalin-fixed tumor tissue sections were incubated in antigen retrieval buffer (Beyotime) in a 100 °C water bath for 20 min. After peroxidase treatment and protein blocking, the tissue sections were incubated with the anti-human PTEN antibody (1:325 diluted with 5% bovine serum albumin) and rabbit polyclonal antibody (Cell Signaling Technology) at 37 °C in a humidified chamber for 1 h. The sections were then visualized by using the SABC kit (Boster, Wuhan, China) according to the manufacturer’s instruction. The sections were counterstained with hematoxylin and eosin. The sections then were photographed with the Nikon Eclipse Ti microscope and NIS elements software (Nikon, Tokyo, Japan). The intensity of IHC staining on sections without the primary antibody was subtracted from that of the corresponding sections with the primary antibody staining to exclude the influence of pigmentation and background staining.

### Luminescent reporter gene transfection and luciferase assays

miRNA targets were predicted by using the miRanda database (http:// http://www.targetscan.org/). For the luciferase activity assay, nucleotides 710 to 730 (the complete predicted miR-486-5p target site) of the *PTEN* 3′-UTR were inserted downstream of the *Renilla* luciferase gene in a *Renilla*/firefly luciferase reporter plasmid, psiCHECK-2 (GenePharma, Shanghai, China). The scramble oligonucleotides (negative control) and hsa-miR-486-5p mimic (miR-486-5p mimic) were synthesized by GenePharma. 293 T cells were transfected with 0.5 μg of reporter plasmids per well plus scramble oligonucleotides or miR-486-5p mimic. Transfection for 24 h later, *Renilla*/firefly luciferase activity was measured by a dual luciferase reporter assay (Promega, Fitchburg, WI, USA) in an automatic microplate reader (Thermo Scientific, Waltham, MA, USA).

### Biostatistical analysis

The one-way ANOVA and Student *t*-tests were used, with the Welch correction for variables, to assess the significance of differences between groups. All statistical analyses used GraphPad 6.0 software. A *P*-value < 0.05 was considered statistically significant.

## Results

### MiR-486-5p is overexpressed in cervical cancer patients’ sera and tissues

**Fig. 1:**
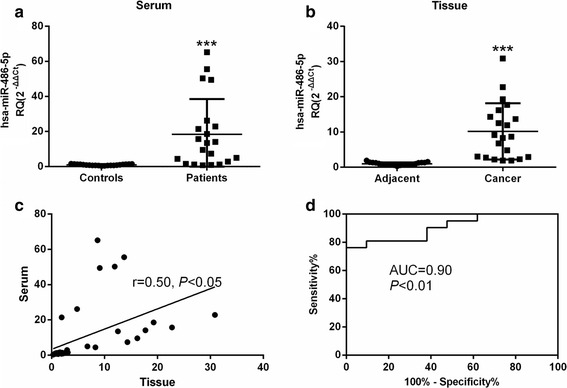
miR-486-5p is overexpressed in serum and tumor tissues from cervical cancer patients. **a** miR-486-5p expression in serum from healthy donors (Controls) and cervical cancer patients (Patients). ***P < 0.001, compared with Controls. **b** miR-486-5p expression in cervical cancer patients’ cancer tissues (Cancer) and adjacent normal tissues (Adjacent). ***P < 0.001, compared with adjacent tissues. **c** The correlation of miR-486-5p expression in cervical cancer patients’ serum and tissues. **d** ROC analysis of the serum miR-486-5p expression assay results for cervical cancer patients vs healthy subjects

miR-486-5p expression was 18.56-fold higher in cervical cancer patients’ sera than in healthy donors’ sera (*P* < 0.001, Fig. 1a) and 10.40-fold higher in tumor tissues from cervical cancer patients than in adjacent normal tissues (*P* < 0.001, Fig. 1b). There was a significant correlation between miR-486-5p expression in serum and in tissues (*r* = 0.50, *P* < 0.05, Fig. 1c). With an AUC = 0.90, serum miR-486-5p qualifies as a diagnostic biomarker for cervical cancer (*P* < 0.01, Fig. 1d).

We assessed the correlation between serum miR-486-5p concentration and four clinicopathologic factors in the cervical cancer patients: age, human papillomavirus infection status, tumor histologic type, and therapies received. In both univariate analysis and multivariate analyses, none of these factors was significantly correlated with higher serum concentration of miR-486-5p in these patients (Table 1). This indicates that miR-486-5p could be an independent biomarker to diagnose cervical cancer.

### MiR-486-5p is expressed in cervical cancer cell lines

**Fig. 2:**
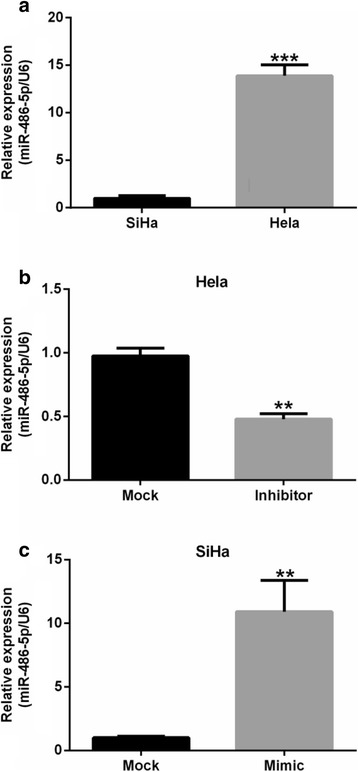
miR-486-5p is expressed in cervical cancer cells. **a** miR-486-5p expression in HeLa cells and SiHa cells. ***P < 0.001, compared to SiHa cells. **b** miR-486-5p expression in HeLa cells transfected with miR-486-5p inhibitor sponge (Inhibitor) or matching control plasmid (Mock). **c** miR-486-5p expression in SiHa cells transfected with miR-486-5p mimic (Mimic) or matching control plasmid (Mock). **P < 0.01, compared with Mock

MiR-486-5p expression was 13.88-fold higher in HeLa cells than in SiHa cells (*P* < 0.001, Fig. 2a). Transfection of plasmids expressing miR-486-5p inhibitor sponge into HeLa cells decreased miR-486-5p expression by 51% compared with cells transfected with the relevant control (Mock) plasmids (*P* < 0.01; Fig. 2b). In contrast, transfection of miR-486-5p mimic into SiHa cells increased miR-486-5p expression by 10.92-fold compared with Mock-transfected controls (*P* < 0.01; Fig. 2c).

### MiR-486-5p increases proliferation, colony formation, migration, and invasion of cervical cancer cells

**Fig. 3:**
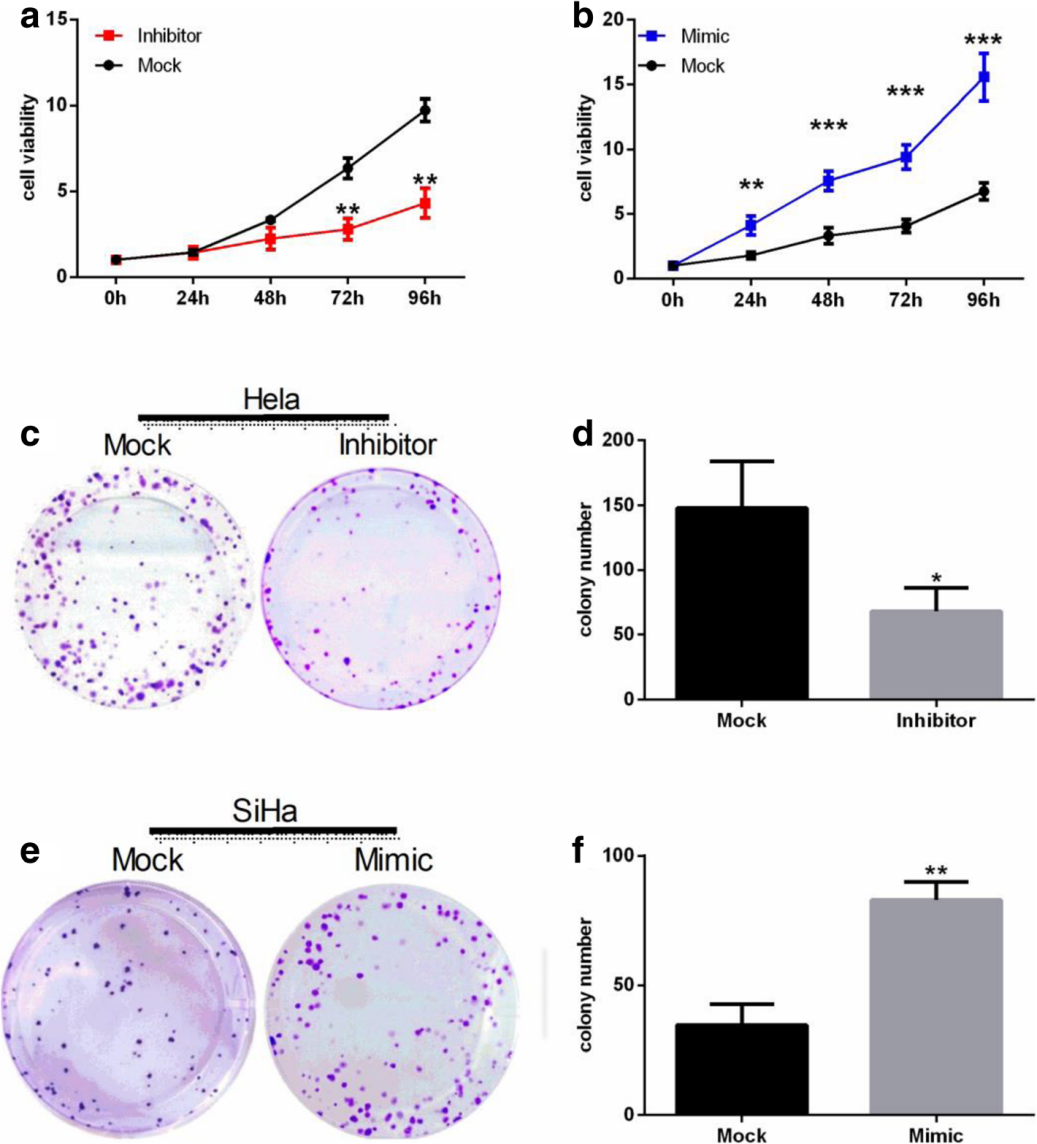
miR-486-5p promotes cervical cancer cell proliferation in vitro. **a** Cell proliferation assay comparing HeLa cells transfected with miR-486-5p inhibitor sponge (*red*) or control miRNA (*black*) for 96 h. **b** Cell proliferation assay comparing SiHa cells transfected with miR-486-5p mimic (*blue*) or control miRNA (*black*) for 96 h. **c**-**d** Colony formation assay: HeLa cells transfected with miR-486-5p inhibitor sponge (Inhibitor) or control miRNA (Mock) were incubated in 6-well culture plates. Colonies were counted after 15 days. **e**-**f** Colony formation assay: SiHa cells transfected with miR-486-5p mimic (Mimic) or control miRNA (Mock) were cultured in 6-well culture plates. Colonies were counted after 15 days. *P < 0.05, ** *P* < 0.01, *** *P* < 0.001, compared with Mock

Proliferation of HeLa cells transfected with miR-486-5p inhibitor was 2%, 33%, 56%, and 56% lower, respectively, at 24 (*P* > 0.05), 48 (*P* > 0.05), 72 (*P* < 0.01), and 96 h (*P* < 0.01) than that of cells transfected with control miRNA (Fig. 3a). In contrast, upregulation of miR-486-5p via miR-486-5p mimic transfection enhanced proliferation of SiHa cells by 2.28-, 2.30-, 2.31-, and 2.31-fold, respectively, at 24 (*P* < 0.01), 48 (*P* < 0.001), 72 (*P* < 0.001), and 96 h (*P* < 0.001; Fig. 3b). In colony formation assays performed to determine cell viability and proliferation activity, the number of colonies formed by HeLa cells transfected with miR-486-5p inhibitor was markedly lower than the number of colonies formed by Mock-transfected control cells (*P* < 0.05; Fig. 3c-d), while the number of colonies formed by miR-486-5p mimic-transfected SiHa cells was significantly greater than that formed by controls (*P* < 0.01; Fig. 3e-f).

**Fig. 4:**
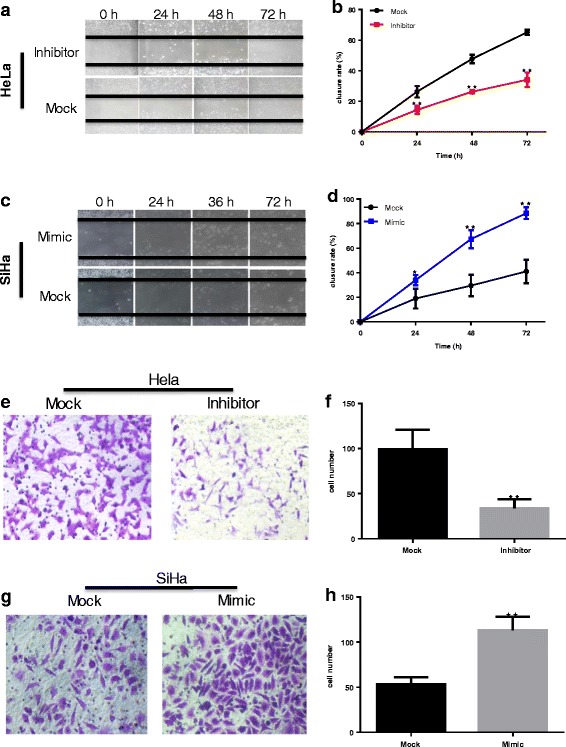
miR-486-5p promotes cervical cancer cell migration and invasion in vitro*.* **a**-**d** Cell migration was assessed by the scratch repair (wound healing) test. Extent of wound closure indicates degree of cell migration. **a**-**b** HeLa cells transfected with miR-486-5p inhibitor sponge (Inhibitor) or control miRNA (Mock) were scratched and incubated in medium with 2% FBS for 72 h. **c**-**d** SiHa cells transfected with miR-486-5p mimic (Mimic) or control miRNA (Mock) were scratched and incubated in medium with 2% FBS for 72 h. **e**-**h** Cell invasiveness was assessed by the transwell assay. Cell invasiveness was measured by determining the number of cells that migrated through a matrigel coating in the transwell chamber. **e**-**f** HeLa cells transfected with miR-486-5p inhibitor sponge (Inhibitor) or control miRNA (Mock) were placed in the transwell chambers and allowed to incubate for 24 h. **g**-**h** SiHa cells transfected with miR-486-5p mimic (Mimic) or control miRNA (Mock) were placed in the transwell chambers and allowed to incubate for 24 h. ** *P* < 0.01, compared with Mock

MiR-486-5p inhibition significantly decreased HeLa cell migration, as determined by a wound closure assay, whereas overexpression of miR-486-5p in SiHa cells significantly increased migration. The wound closure rates of miR-486-5p inhibitor–transfected HeLa cells were 11.91%, 21.48%, and 31.04% lower 24 h, 48 h, and 72 h after scratching, respectively, than those of cells transfected with Mock control plasmid (*P* < 0.01, *P* < 0.01, and *P* < 0.01, respectively; Fig. 4a-b). The wound closure rates of miR-486-5p mimic–transfected SiHa cells 24 h, 48 h, and 72 h after scratching were 14.91%, 37.81%, and 47.38% higher, respectively, than those of cells transfected with Mock control plasmid (*P* < 0.05, *P* < 0.01, and *P* < 0.01, respectively; Fig. 4c-d). Similarly, the invasiveness of miR-486-5p inhibitor–transfected HeLa cells on the transwell assay was significantly lower than that of Mock-transfected control cells (*P* < 0.01; Fig. 4e-f), whereas the invasiveness of miR-486-5p mimic–transfected SiHa cells was significantly greater than that of controls (*P* < 0.01; Fig. 4g-h).

### MiR-486-5p activates the PI3K-Akt pathway by directly inhibiting *PTEN*

**Fig. 5:**
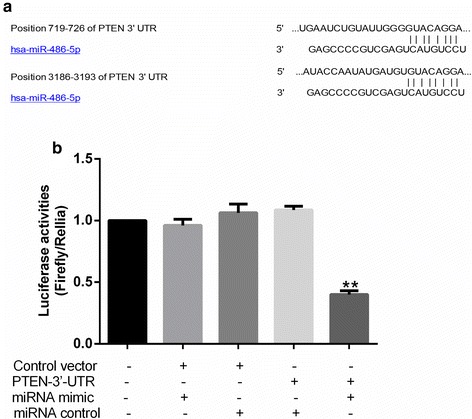
miR-486-5p directly targets PTEN. **a** Sequence alignment of miR-486-5p and its conserved target site in the *PTEN* 3′-UTR. **b** Luciferase activity was measured in 293 T cells with a dual luciferase reporter assay. The cells were co-transfected with a plasmid expressing miR-486-5p mimic or control miRNA and a vector expressing PTEN 3′-UTR or empty vector. Firefly luciferase activity was normalized to *Renilla* luciferase activity, and the value for the empty vector was used as the control. **P < 0.01, compared with all other groups

To understand the mechanisms underlying the effects of miR-486-5p on cervical cancer, we examined the potential target of miR-486-5p by in silico analysis using the TargetScanHuman6.2 database (http://www.targetscan.org/). This analysis identified *PTEN* as a candidate target of miR-486-5p, with two binding sites (Fig. 5a). To demonstrate miR-486-5p directly bund to this gene, we co-transfected miR-486-5p mimic, control miRNA, or control plasmid with a vector expressing *PTEN* luc-3′-UTR or empty vector into 293 T cells, then caculated their luciferase activity. Cells transfected with miR-486-5p mimic had 60% lower luciferase activity than the control plasmid (*P* < 0.01; Fig. 5b). This suggested that *PTEN* expression was suppressed by miR-486-5p binding to its 3′-UTR. The binding was specific, because the luc-reporter vector was not affected by the control miRNA, and the control vector was not affected by the miR-486-5p mimic (*P* = 0.64; Fig. 5b).

**Fig. 6:**
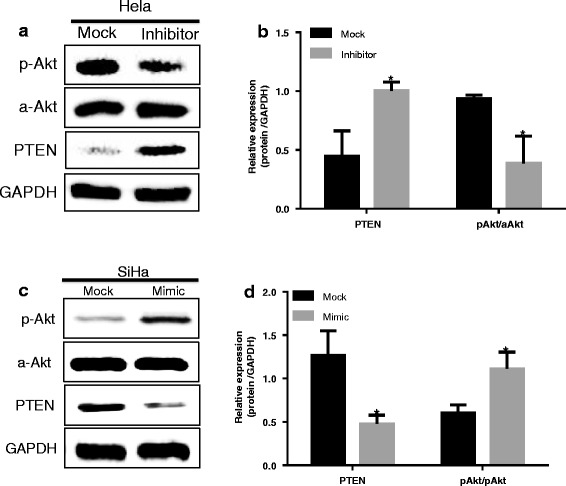
miR-486-5p regulates expression of PTEN and PI3K/AKT pathway proteins. Levels of the indicated PI3K/AKT pathway proteins were analyzed in cervical cancer cells by western blotting; differences in expression are represented in the bar graphs. **a**-**b** HeLa cells were transfected with miR-486-5p inhibitor sponge (Inhibitor) or control miRNA (Mock). **c**-**d** SiHa cells were transfected with miR-486-5p mimic (Mimic) or control miRNA (Mock). Data (mean±SD; *n* = 3) and images shown are representative of one experiment. Similar results were obtained in three independent experiments. *P < 0.05, compared with Mock

To demonstrate the role of miR-486-5p in activating the PI3K/PTEN/Akt pathway, we analyzed the protein levels of PTEN, phosphorylated Akt (p-Akt), and all-Akt in cervical cancer cells by western blotting. In HeLa cells transfected with miR-486-5p inhibitor, expression of PTEN was significantly increased and expression of p-Akt was significantly decreased compared with that in Mock-transfected control cells (all, *P* < 0.05; Fig. 6a-b). In contrast, in SiHa cells transfected with miR-486-5p mimic, expression of PTEN was significantly decreased and expression of p-Akt was highly increased compared with that in the control cells (all, *P* < 0.05; Fig. 6c-d).

### Downregulation of miR-486-5p inhibits tumor growth in a cervical cancer xenograft mouse model

**Fig. 7:**
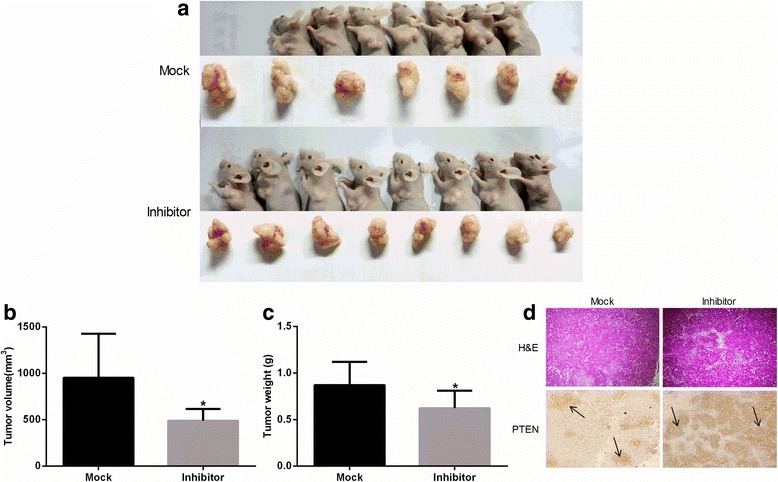
Downregulation of miR-486-5p inhibits cervical cancer growth in vivo by targeting PTEN. The cervical cancer mouse model was constructed by injecting mice with HeLa cells transfected with control miRNA (Mock) or miR-486-5p inhibitor sponge (Inhibitor). **a** Mice were sacrificed on day 21 and their tumors removed and weighed and measured. **b** The volumes of the tumors from the two groups were calculated and compared. The tumor volume was calculated with the following formula: (Length × Width^2^) × 3.14/6. **c** The weights of the tumors from the two groups were compared. **d** The expression of PTEN in local tumor tissues was determined by immunohistochemistry (40×). Controls were stained with hematoxylin and eosin (H&E). The *arrows* show PTEN expression in local tumor tissues. *P < 0.05, compared to Mock

To investigate the effect of miR-486-5p on cervical cancer tumor growth in vivo, HeLa cells transfected with miR-486-5p inhibitor or mock miRNA were inoculated into the left axilla of nude mice. As expected, the tumors that developed from the miR-486-5p–downregulated cells had significantly lower volumes and weights than those that formed from controls 4 weeks after inoculation (Fig. 7a-c). In addition, IHC analysis showed that PTEN expression was markedly higher in the tumors formed from miR-486-5p inhibitor–transfected cells than in the tumors formed from control cells (Fig. 7d).

## Discussion

This study characterized miR-486-5p as an oncogenic miRNA that is highly expressed in and associated with cervical cancer. Suppression of miR-486-5p expression significantly inhibited proliferation, colony formation, migration, and invasion of one cervical cancer cell line (HeLa) in vitro and inhibited tumor growth in a mouse HeLa xenograft model. Overexpression of miR-486-5p in another cervical cell line (SiHa) stimulated cell proliferation, colony formation, migration, and invasion in vitro.

The clinical significance of miR-486-5p in cancer remains largely unknown. Some reports suggest that miR-486-5p is a tumor suppressor in lung cancer [14] and gastric adenocarcinoma [13], while others indicate that it is oncogenic in myeloid leukemia of Down syndrome [11] and chronic myeloid leukemia [12]. Wang et al. found that miR-486-5p expression was decreased in non-small cell lung cancer (NSCLC) tumor tissue and that downregulation of miR-486-5p promoted tumor progression and migration [14]. In contrast, Li et al. demonstrated that miR-486-5p was increased in NSCLC patients’ serum and tumor tissue and that downregulation of miR-486-5p prolonged recurrence-free survival [16].

Because there has been no study on miR-486-5p function in cervical cancer, our purpose here was to investigate the biological functions of this miRNA and their potential underlying mechanisms in this disease. Our results show that expression of miR-486-5p in both serum and tumor tissue is increased in cervical cancer patients and that miR-486-5p expression level regulates cell proliferation, migration, and invasion of cervical cancer cells in vitro and tumor growth in vivo. We conclude from these results that miR-486-5p functions as an oncogenic miRNA in cervical cancer. Its biological functions are mediated through *PTEN,* which was confirmed as a candidate target of miR-486-5p by the TargetScan database. This was further confirmed by a dual luciferase reporter assay whose results show that miR-486-5p directly targeted the *PTEN* 3′-UTR. Previous studies have shown that activation of miR-486-5p blocked PTEN, thus leading to Akt phosphorylation in both mouse and human [17, 18]. Both mouse and human have the same sequences of mature miR-486-5p and *PTEN* 3′-UTR. We showed that inhibition of miR-486-5p upregulated expression of the PTEN protein in HeLa cells, while overexpression of miR-486-5p downregulated PTEN protein expression in SiHa cells. In our in vivo studies, knockdown of miR-486-5p increased PTEN expression in tumor tissue.

## Conclusions

We demonstrate for the first time that serum miR-486-5p has diagnostic potential in cervical cancer. Our results also reveal miR-486-5p to be an oncogenic miRNA that stimulates cell proliferation, migration, invasion, and tumor growth in cervical cancer. The tumorigenic activity of miR-486-5p is mediated through inhibition of *PTEN* expression and activation of the oncogenic PI3K/Akt pathway.

## Abbreviations

Akt: Protein kinase B
FBS: Fetal bovine serum
miR-486-5p: microRNA-486-5p
PBS: Phosphate-buffered saline solution
PI3K: Phosphatidyl Inositol 3-kinase
PTEN: Phosphatase and tensin homolog
RT-PCR: Real-time quantitative polymerase chain reaction
